# Extraskeletal Ewing Sarcoma: A Case Report

**DOI:** 10.7759/cureus.55077

**Published:** 2024-02-27

**Authors:** Ryan Denis, Martin Felix, Daniela Mejia, Mikayla Hobbs, Paul Alvarez, Damian Casadesus

**Affiliations:** 1 Internal Medicine, St. George’s University School of Medicine, St. George, GRD; 2 Internal Medicine, Jackson Memorial Hospital, Miami, USA

**Keywords:** extraskeletal ewing sarcoma, ewing sarcoma (es), ­skin cancer, cutaneous oncology, sarcoma soft tissue

## Abstract

Ewing sarcoma is one of the most common primary bone tumors arising from neuroectodermal cells mainly presenting in the younger population. Instances of this highly malignant tumor manifesting outside of the bone and outside of the typical age range create an unfamiliar clinical scenario. In this report, we present a rare extraskeletal Ewing sarcoma in a 42-year-old woman with a subcutaneous soft tissue mass in the posterior chest displaying a positive *EWSR1 *gene rearrangement via fluorescence in situ hybridization. The patient is currently on a chemotherapy regimen showing favorable response to the tumor size despite additional complications. This overall presentation of Ewing sarcoma allows further understanding of the malignancy and fosters better care for future cases.

## Introduction

Ewing sarcoma (ES), a rare and aggressive form of primary bone cancer, predominantly affects children and young adults. It is characterized by small round cells and comprises 10-15% of all bone sarcomas. This malignancy arises from primitive neuroectodermal cells and commonly manifests within the bone marrow of the long bones, pelvis, and ribs [1]. The peak incidence is typically between 10 and 15 years of age, with around 30% of the cases arising in adults over the age of 20 [1].

However, instances of extraskeletal ES (EES), where the tumor arises outside of the bone, are exceedingly rare and account for 20-30% of all reported cases of ES [2]. EES is characterized by its aggressive nature and tendency to metastasize, posing unique diagnostic and therapeutic challenges when located in soft tissues or other extramedullary sites. For extraskeletal primary tumors, the most common sites of the disease include the trunk (32%), extremity (26%), head and neck (18%), retroperitoneum (16%), and other sites (9%) [3].

The rarity of EES necessitates a meticulous examination of individual cases to enhance our understanding of its clinical behavior, optimal diagnostic approaches, and tailored therapeutic interventions. Our patient’s clinical presentation, diagnostic workup, and treatment course offer valuable insights into the challenges faced by clinicians when confronted with this atypical manifestation of EES. Through the exploration of clinical nuances and therapeutic interventions, this case report seeks to enhance our understanding of EES and ultimately improve the care and prognosis for individuals facing this challenging diagnosis.

## Case presentation

**Figure 1:**
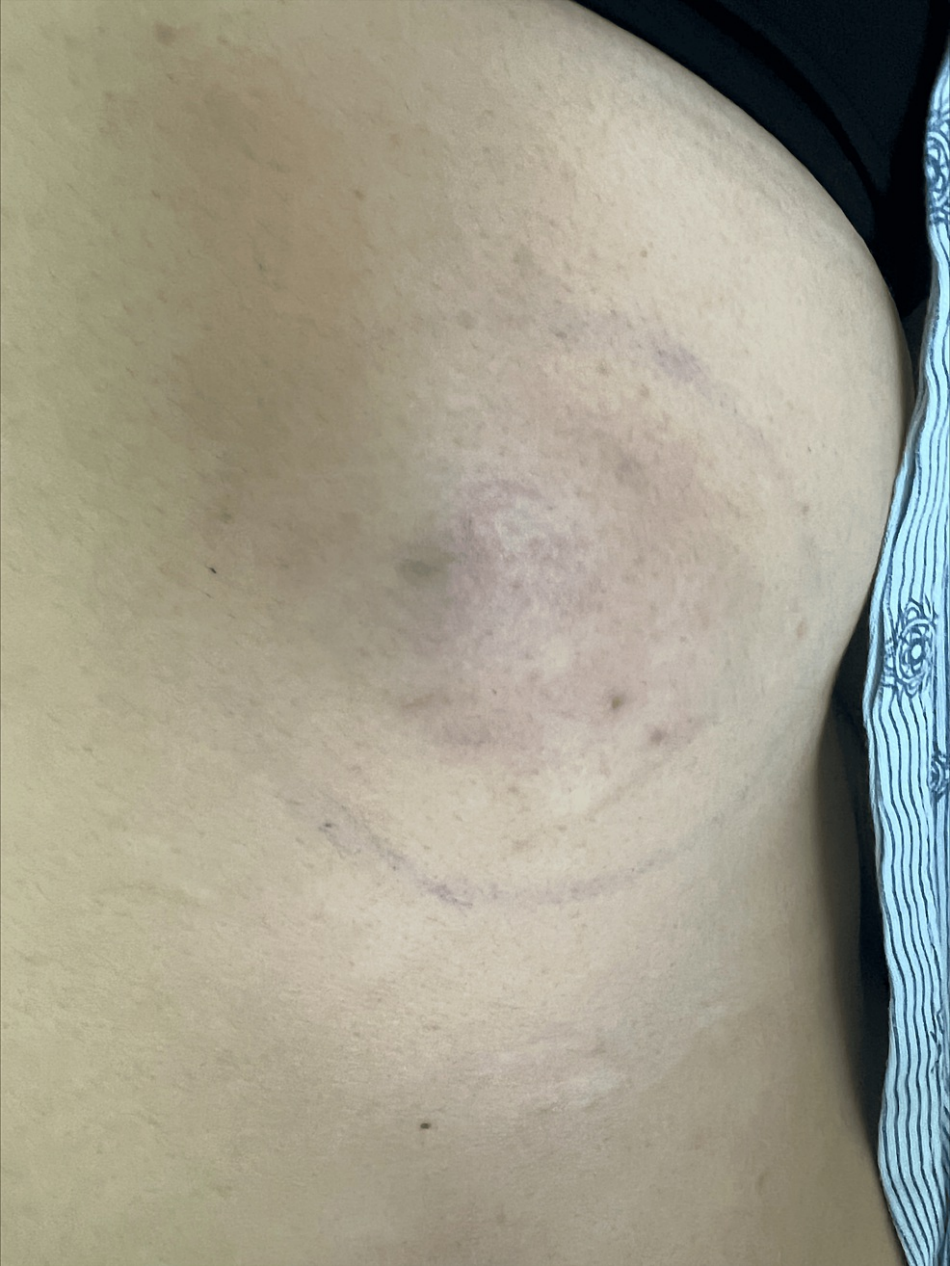
Subcutaneous mass in the posterolateral right side of the chest.

A 42-year-old Hispanic woman presented to the emergency department (ED) with a one-day history of worsening right-sided back pain. She had a past medical history of autosomal dominant polycystic kidney disease, hypertension, and meningioma. A month before this visit, she visited the ED with a similar presentation of back pain and complaints of a growing mass in the back. In the ED, vital signs were within normal limits. On physical examination, the patient had a mobile, subcutaneous, right-sided, posterolateral chest mass measuring 4 x 4 cm that was hard on palpation with minimal skin changes (Figure 1).

**Figure 2:**
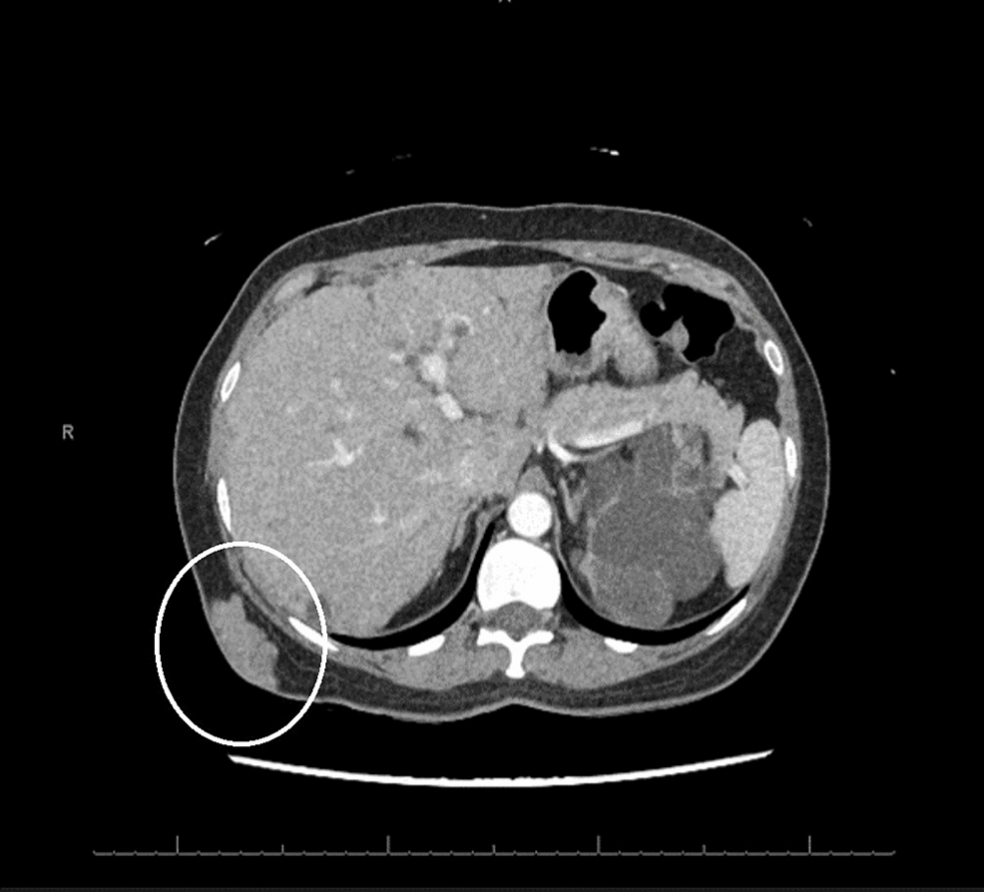
Computed tomography scan with intravenous contrast revealing a soft tissue mass in the subcutaneous fat of the right posterolateral chest wall.

**Figure 3:**
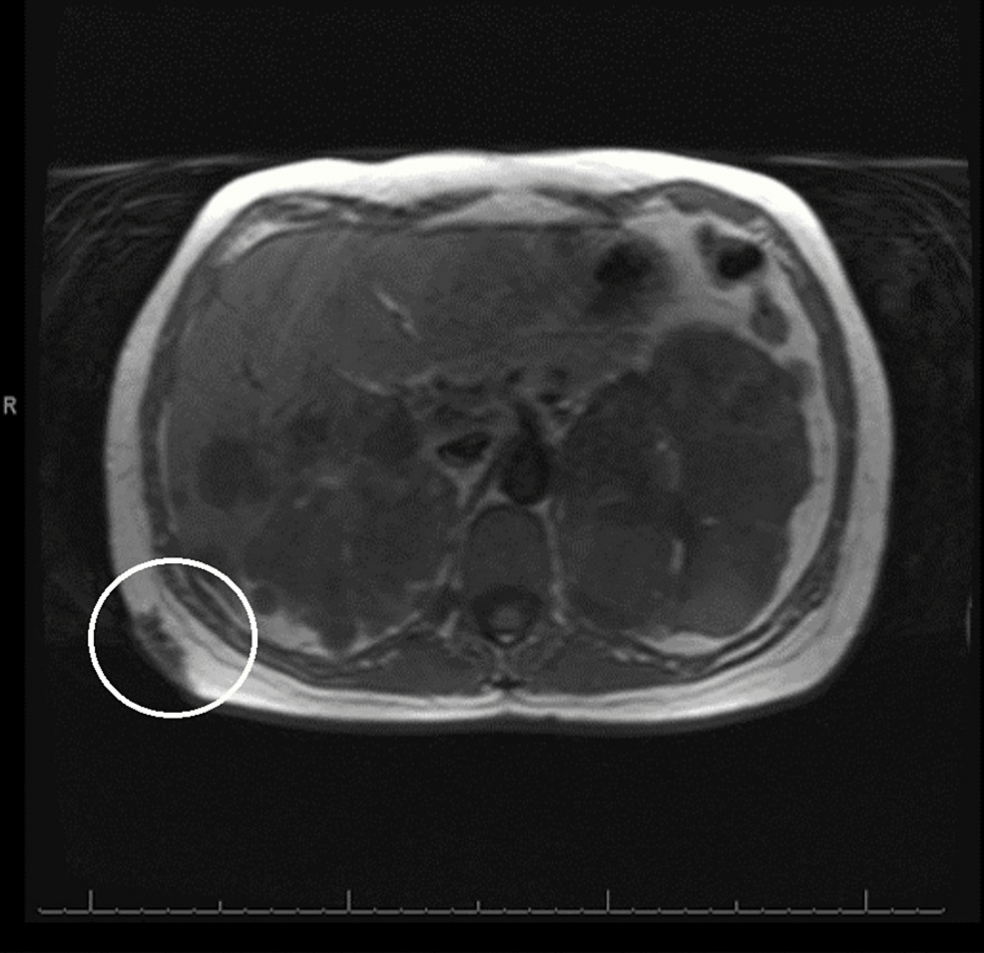
Magnetic resonance imaging of the chest with and without intravenous contrast revealing evidence of polycystic kidney disease affecting the kidneys and liver and a mass in the right-sided posterior chest.

Imaging with an abdominal and pelvis computed tomography (CT) scan with intravenous (IV) contrast demonstrated a stable soft tissue mass in the subcutaneous fat of the right posterior lateral chest wall (Figure 2). Magnetic resonance imaging (MRI) of the chest with and without IV contrast revealed evidence of polycystic kidney disease affecting the kidneys and liver and a mass in the right-sided posterior chest (Figure 3). The MRI with and without IV contrast did not show any evidence of possible metastatic disease in the thoracic or lumbar spine.

**Figure 4:**
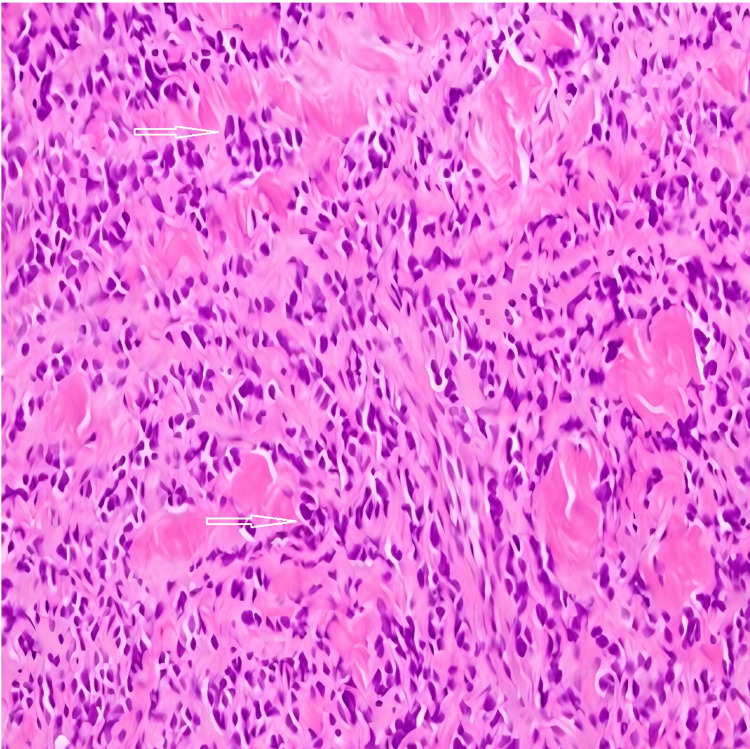
Infiltrating tumor in the dermis composed of small round blue cells with minimal cytoplasm and nuclear overlapping (hematoxylin and eosin, 200×).

**Figure 5:**
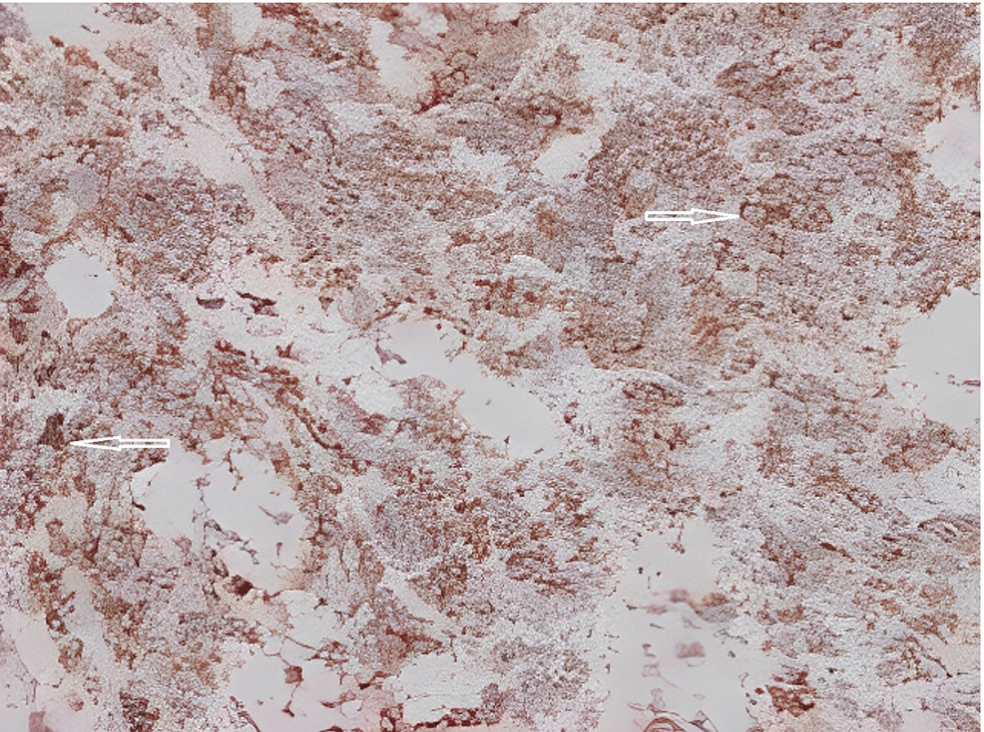
CD99 immunostaining (a sensitive but not specific marker for Ewing sarcoma) showing positive membranous staining (200×).

**Figure 6:**
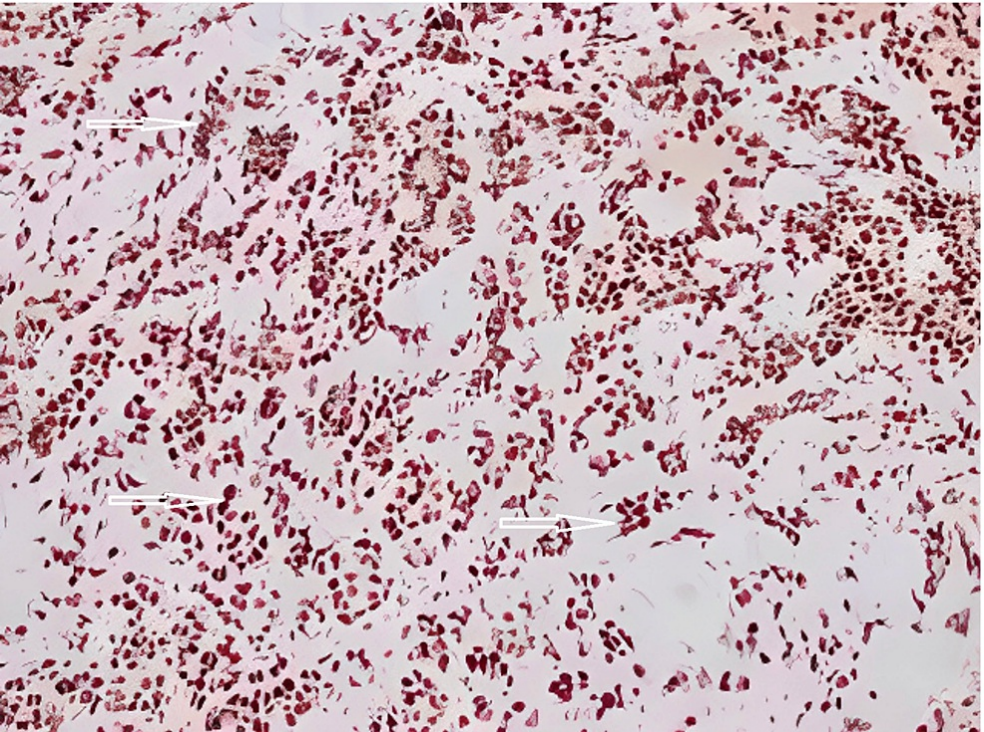
NKX2.2 immunostaining (sensitive and specific for Ewing sarcoma) showing positive nuclear staining (200×).

The patient underwent a core needle biopsy. The histopathologic study revealed atypical small round cells consistent with ES (Figures 4-6). The tumor was found to be positive for CD99 and NKX2.2 and negative for AE1/AE3, CK8/CK18, CK7, CK20, desmin, SOX10, BCOR, WT1, CD45, CD31, and merkel antigen, supporting the diagnosis of EES. Further tissue studies detected an* EWSR1* signal in 80% of nuclei, indicating the presence of an *EWSR1 *rearrangement. Other images did not reveal metastatic disease at the time of the diagnosis of the tumor.

The patient started treatment with a systemic chemotherapy regimen with vincristine, ifosfamide, and doxorubicin. The patient’s course was complicated with neutropenic fever secondary to a vestibular abscess of tooth #19, treated with extraction and amoxicillin-clavulanate. After the first round of chemotherapy, the patient had a discernible response with shrinkage in the size of the tumor.

## Discussion

ES of the bone (ESB) is a predominantly pediatric primary malignancy and 10 times more common than EES. The latter type of sarcoma has an incidence of 0.4 per million and is most common in patients below the age of 5 and above the age of 35 and in women [4-6]. On presentation, patients with EES most often report pain in the upper regions of the arms, shoulders, buttocks, and thighs, with 25% of cases involving bone marrow, lung, and bone metastases. Our patient’s presentation is very interesting because the tumor presented at an older age and in the subcutaneous tissue.

The most used imaging modalities for the diagnosis of EES include MRI, CT scan, and ultrasonography. In our patient, the imaging studies revealed the key findings reported in the literature, including a stable, hyperlobulated, distinct borders, and hyperdense lesion located in the subcutaneous fat; however, no areas of necrosis were observed [7-9]. No evidence of metastatic disease in the lungs and bones was observed in our patient. The typical histological findings of clusters of blue small and round with cytoplasmic borders and nucleoli that are difficult to delineate from nearby structures and large and round nuclei were present in our patient [10]. Other studies have revealed positive CD99 and NKX2.2, as well as an abnormal fluorescence in situ hybridization result demonstrating an *EWSR1* rearrangement in 80% of cells.

There is currently a very limited amount of information in the literature regarding the best form of treatment for EES; however, surgery with or without radiotherapy, along with chemotherapy, has been described as the most viable option [11,12]. ES of the skin appears to have a more favorable prognosis, with 91% of the patients surviving at 10 years which is higher than ESB [13,14]. Current recommendations endorse a two-week, rather than a three-week, administration interval of vincristine-doxorubicin, ifosfamide-etoposide, and cyclophosphamide cycles, with a maximum event-free survival rate of about 73% [15].

Studies have described fewer than 100 cases of EES in the literature. The tumor is more frequent in white women and the pediatric population and the presence of metastasis is very rare at the time of diagnosis. Our patient, in the fourth decade of life, presented without metastatic disease, and after the treatment, the tumor size improved [13,16-18].

## Conclusions

EES is associated with an indolent course, rarely has metastatic disease at the time of diagnosis, and has a favorable prognosis when treated with combined modality therapy. Less invasive chemotherapy should be considered a potential treatment for EES because of the low rate of metastatic disease at diagnosis, local control, good response to treatment, and excellent outcome, as well as decreased side effects and toxicity with a high curative rate. Physicians should consider ES in the differential diagnosis of subcutaneous tumors, even in older age.
